# Supplementary material for: Relationships between topographic factors, soil and plant communities in a dry Afromontane forest patches of Northwestern Ethiopia
Source: PLoS One. 2021 Mar 12;16(3):e0247966. doi: 10.1371/journal.pone.0247966 (PMC7954303; doi:10.1371/journal.pone.0247966)
Supplement: S1 Appendix — (DOCX) [file pone.0247966.s001.docx]

**S1 Appendix.** Floristic List of Dega Damot district

| **No** | Species | Family | Habit | Local name |
| --- | --- | --- | --- | --- |
| 1 | *Abutilon figarianum* Webb | Malvaceae | Herb | Nacha |
| 2 | *Acacia abyssinica* Hochst. ex Benth. | Fabaceae | Tree | Girar |
| 3 | *Acaciaetbaica*Schweinf. | Fabaceae | Tree | Korara girar |
| 4 | *Acacia seyal* Del. | Fabaceae | Tree | Nech Girar |
| 5 | *Acanthus sennii*Chiov. | Acanthaceae | Shrub | Kosheshila |
| 6 | *Achyranthes aspera*L. | Amaranthaceae | Herb | - |
| 7 | *Achyrospermum schimperi*(Hochst. ex Briq.)Perkins | Lamiaceae | Herb | - |
| 8 | *Acokanthera schimperi* (A.DC.) Schweinf | Apocynaceae | Tree | Merenz |
| 9 | *Albizia schimperiana*Oliv. | Fabaceae | Tree | - |
| 10 | *Allophylus abyssinicus*(Hochst.) Radlk. | Sapindaceae | Tree | Embis |
| 11 | *Aloe pulcherrima* Gilbert & Sebsebe | Aloaceae | Shrub | Eret |
| 12 | *Andropogon abyssinicus*Fresen. | Poaceae | Herb | Gaja Sar |
| 13 | *Anthospermum herbaceum*L.f. | Rubiaceae | Herb | - |
| 14 | *Apodytes* dimidiata E. Mey. ex Arn.acutifolia (Hochst. ex A. Rich.) Boutique | Icacinaceae | Tree | Dong |
| 15 | *Arisaema enneaphyllum*Hochst. ex A.Rich. | Araceae | Herb | Amoch |
| 16 | *Arundinaria alpina*K. Schum. | Poaceae | Tree | Kerkeha |
| 17 | *Arundo donax* L. | Poaceae | Shrub | Shembeqo |
| 18 | *Asparagus africanus* Lam. | Asparagaceae | Liana | - |
| 19 | *Bersama abyssinica* Fresen. subsp. *abyssinica* | Melianthaceae | Shrub | Azamara |
| 20 | *Berula erecta* (Hudson) Coville | Apiaceae | Herb | - |
| 21 | *Bidens macroptera* (Sch. Bip. ex Chiov.) Mesfin | Asteraceae | Herb | AdeyAbaba |
| 22 | *Bothriocline schimperi*Sch. Bip. ex Walp. | Asteraceae | Shrub | - |
| 23 | *Bruceaantidysenterica* J. F. Mill | Simaroubaceae | Shrub | Abalo |
| 24 | *Buddleja davidii* Franch. | Loganiaceae | Shrub | Tikuir Anfar |
| 25 | *Buddleja polystachya* Fresen. | Loganiaceae | Shrub | Nech anfar |
| 26 | *Calpurnia aurea*(Ait.) Benth. | Fabaceae | Shrub | Ligita |
| 27 | *Carduus nyassanus* (S. Moore) R.E. Fr. | Asteraceae | Herb | - |
| 28 | *Carduus schimperi* Sch. Bip. | Asteraceae | Herb | Yemidir eshoh |
| 29 | *Carissa spinarum* L. | Apocynaceae | Shrub | Agam |
| 30 | *Cassipourea malosana* (Baker) Alston | Rhizophoraceae | Tree | Tikur Enchet |
| 31 | *Catha edulis* (Vahl) Forssk. ex Endl. | Celastraceae | Shrub | chat |
| 32 | *Clausena anisata*(Willd.) Benth. | Rutaceae | Shrub | Limich |
| 33 | *Clematis simensis* Fresen. | Ranunculaceae | Liana | Azo hareg |
| 34 | *Clerodendrum myricoides* (Hochst.) Vatke | Lamiaceae | Shrub | - |
| 35 | *Clutia abyssinica* Jaub. & Spach. | Euphorbiaceae | Shrub | Fiyelfej |
| 36 | *Combretum collinum* Fresen. | Combretaceae | Tree | Wonde Abalo |
| 37 | *Combretum molle* R. Br. ex G. Don | Combretaceae | Tree | Sete Abalo |
| 38 | *Commelina benghalensis* L. | Commelinaceae | Herb | Eregna kolw |
| 39 | *Convolvulus kilimandschari* Engl. | Convolvulaceae | Liana | - |
| 40 | *Crassocephalum macropappum* (Sch. Bip. ex A. Rich.) S. Moore | Asteraceae | Herb | - |
| 41 | *Crotalaria rosenii* (Pax) Milne-Redh. ex Polhill | Fabaceae | Shrub | - |
| 42 | *Croton macrostachyus* Del. | Euphorbiaceae | Tree | Bisana |
| 43 | *Cynoglossum coeruleum* Hochst. ex A.DC. | Boraginaceae | Herb | Shingug |
| 44 | *Cyperus dereilema* Steud. | Cyperaceae | Herb | Giramita |
| 45 | *Cyperus squarrosus* L*.* | Cyperaceae | Herb | Ketema sar |
| 46 | *Cyphostemma cyphopetalum* (Fresen.) Desc. ex Wild& Drummond | Vitaceae | Herb | - |
| 47 | *Datura stramonium* L. | Solanaceae | Herb | Astenagir |
| 48 | *Delphinium* wellbyi Hemsl. | Ranunculaceae | Herb | Gedel Amuk |
| 49 | *Desmodium velutinum*(Willd.) DC. | Fabaceae | Herb | - |
| 50 | *Dicranopteris linearis* (Burm. f.) Underw. | Gleicheniaceae | Herb | Fern |
| 51 | *Dipsacus pinnatifidus* Steud. ex A. Rich. | Dipsacaceae | Herb | Enkelekey |
| 52 | *Discopodium penninervium* Hochst. | Solanaceae | Tree | Aluma |
| 53 | *Dodonaea angustifolia*L. f | Sapindaceae | Shrub | Kitkita |
| 54 | *Dombeyatorrida* (J.F.Gmel.) P.Bamps | Sterculiaceae | Tree | Wilkifa |
| 55 | *Dovyalis verrucosa* (Hochst.) Warb. | Flacourtiaceae | Shrub | Koshim |
| 56 | *Drynaria volkensii* Hieron. | Polypodiacie | Herb | Fern spp |
| 57 | *Echinops pappii Chiov.* | Asteraceae | Shrub | Ahiya eshoh |
| 58 | *Ekebergia capensis*Sparrm. | Meliaceae | Tree | Lol |
| 59 | *Embelia schimperi* Vatke | Myrsinaceae | Liana | Enkoko |
| 60 | *Ensete ventricosum* (Welw.) Cheesman | Musaceae | Herb | Enset |
| 61 | *Entada abyssinica* Steud. ex A. Rich. | Fabaceae | Tree | - |
| 62 | *Epilobium hirsutum* L. | Onagraceae | Herb | - |
| 63 | *Erica arborea* L. | Ericaceae | Shrub | Asta |
| 64 | *Erythrina brucei* Schweinf. | Fabaceae | Tree | Korch |
| 65 | *Eucalyptus globulus* Labill | Myrtaceae | Tree | Bahirzaf |
| 66 | *Euclea schimperi*(A. DC.) Dandy | Ebenaceae | Shrub | Dedeho |
| 67 | *Euphorbia abyssinica* Gmel. | Euphorbiaceae | Tree | Kulkual |
| 68 | *Euphorbia schimperiana* Scheele | Euphorbiaceae | Herb | Yegede wetet |
| 69 | *Euphorbia tirucalli* L. | Euphorbiaceae | Shrub | kinchib |
| 70 | *Ferula communis* L. | Apiaceae | Herb | - |
| 71 | *Festuca macrophylla*Hochst. ex A.Rich. | Poaceae | Herb | - |
| 72 | *Ficus ingens* (Miq.) Miq. | Moraceae | Tree | - |
| 73 | *Ficus sur* Forssk. | Moraceae | Tree | Shola |
| 74 | *Ficus thonningii*Blume | Moraceae | Tree | chebeha |
| 75 | *Ficus vasta* Forssk | Moraceae | Tree | Warka |
| 76 | *Galiniera saxifraga*(Hochst.) Bridson | Rubiaceae | Tree | Yetotakolet |
| 77 | *Galium simense* Fresen. | Rubiaceae | Herb | Ashekit |
| 78 | *Geranium ocellatum* Cambess | Geraniaceae | Herb | - |
| 79 | *Girardinia bullosa*(Stedudel) Wedd. | Lamiaceae | Herb | - |
| 80 | *Gladiolus a byssinieus* (Brongn. ex Lemaire) Goldblatt &de Vos | Iridaceae | Herb | yejib Ageda |
| 81 | *Gnidia glauca*(Fresen.)Gilg | Thymelaeaceae | Shrub | Awra |
| 82 | *Grewia ferruginea*Hochst. ex A. Rich. | Tiliaceae | Shrub | Kawt |
| 83 | *Guizotia scabra* (Vis.) Chiov. | Asteraceae | Herb | Mech |
| 84 | *Gymnema sylvestre* (Retz.) R. Br. ex Schult. | Asclepiadaceae | Liana | Yedega medir |
| 85 | *Hagenia abyssinica* (Bruce) J.F. Gmel. | Rosaceae | Tree | Koso |
| 86 | *Helichrysum citrispinum* Del. | Asteraceae | Shrub | Chifrg |
| 87 | *Helichrysum nudifolium*(L.) Less. | Asteraceae | Herb | - |
| 88 | *Helichrysum schimperi* (Sch. Bip. ex A. Rich.) Moeser | Asteraceae | Herb | - |
| 89 | *Helichrysum splendidum* (Thunb.) Less. | Asteraceae | Herb | - |
| 90 | *Hymenodictyon floribundum* (Hochst. & Steud.) Robinson | Rubiaceae | Tree | Gedel Amuk |
| 91 | *Hypericum quartinianum*A. Rich. | Hypericaceae | Shrub | Amija |
| 92 | *Hypericum revolutum*Vahl. | Hypericaceae | Shrub | Amija |
| 93 | *Hypoestes forskaolii*(Vahl) R. Br. | Acanthaceae | Herb | Yebrbere kitel |
| 94 | *Impatiens hochstetteri* Warb. subsp. *hochstetteri* | Balsaminaceae | Herb | Gishirt |
| 95 | *Impatiens rothii* Hook. f. | Balsaminaceae | Herb | Gishirt |
| 96 | *Inula confertiflora*A. Rich. | Asteraceae | Herb | - |
| 97 | *Jasminum abyssinicum* Hochst. ex DC. | Oleaceae | Liana | Abita hareg |
| 98 | *Juniperus procera* L. | Cupressaceae | Tree | Tsid |
| 99 | *Justicia heterocarpa*T. | Acanthaceae | Herb | - |
| 100 | *Kalanchoe petitiana*A. Rich. | Crassulaceae | Herb | Andawila |
| 101 | *Kniphofia foliosa* Hochst. | Asphodelaceae | Herb | Ashegidye |
| 102 | *Lactuca inermis* Forssk. | Asteraceae | Herb | - |
| 103 | *Laggera tomentosa* Sch.-Bip. | Asteraceae | Shrub | Gimane |
| 104 | *Leonotis ocymifolia* (Burm.f.) Iwarsson | Lamiaceae | Shrub | Ras kimir |
| 105 | *Lepidotrichiliavolkensii*(Gurke) Leroy | Meliaceae | Tree | Abalat |
| 106 | *Lippia adoensis* Hochst. ex Walp. var*. adoensis* | Verbenaceae | Shrub | Kesy |
| 107 | *Lobelia giberroa* Hemsl. | Lobeliaceae | Shrub | Gibira |
| 108 | *Lunathyrium boryanum* (Willd.) H. Ohba | Woodsiaceae | herb | - |
| 109 | *Maesa lanceolata* Forssk. | Myrsinaceae | Shrub | Kilaba |
| 110 | *Malva verticillata* L. | Malvaceae | Herb | Tulit |
| 111 | *Maytenus arbutifolia* (A.Rich.) Wilczek | Myrsinaceae | Shrub | Atat |
| 112 | *Maytenus obscura* (A.Rich.) Cuf. | Celastraceae | Tree | Qoba |
| 113 | *Mimusops kummel* A. DC. | Sapotaceae | Tree | Ishe |
| 114 | *Momordica foetida* Schumach. | Cucurbitaceae | Herb | - |
| 115 | *Myrica salicifolia*A.Rich | Myricaceae | Tree | Shi**n**et |
| 116 | *Myrsine africana* L. | Myrsinaceae | Shrub | Kechemo |
| 117 | *Olea capensis*L.subsp. *macrocarpa* (C.H. Wright) Verdc. | Oleaceae | Tree | wegeda |
| 118 | *Olea europaea* L. subsp. *cuspidata* (Wall. ex G.Don) Cif. | Oleaceae | Tree | woyra |
| 119 | *Olinia rochetiana* A. Juss | Oliniaceae | Tree | Tife |
| 120 | *Oplismenus hirtellus* (L.) P. Beauv. | Poaceae | Herb | Yekok Sar |
| 121 | *Osyris quadripartita* Decn. | Santalaceae | Shrub | Qeret |
| 122 | *Otostegia integrifolia* Benth | Lamiaceae | Shrub | Tunjit |
| 123 | *Pennisetum sphacelatum*(Nees) Th. Dur. & Schinz | Poaceae | Herb | Sebez |
| 124 | *Peperomia abyssinica* Miq. | Piperaceae | Herb | - |
| 125 | *Periploca linearifolia* Quaart.-Dill. & A. Rich. | Asclepiadaceae | Liana | Yekola medir |
| 126 | *Phragmanthera regularis* (Sprague) M. Gilbert. | Loranthaceae | Liana | Teketila |
| 127 | *Phytolacca dodecandra*L 'Herit. | Phytolaccaceae | Liana | Endod |
| 128 | *Pittosporium viridiflorum* Sims | Pittosporaceae | Tree | weylwiha |
| 129 | *Plectranthus assurgens* (Baker) J.K. Morton | Lamiaceae | Herb | - |
| 130 | *Plectranthus longipes*Baker | Lamiaceae | Herb | yedimet Ayn |
| 131 | *Pleopeltis macrocarpa* (Willd.) Kaulf | Polypodiaceae | Herb |  |
| 132 | *Premna schimperi* Engl. | Lamiaceae | Shrub | Chafie |
| 133 | *Prunus africana* (Hook. f.) Kalkm. | Rosaceae | Tree | Koma |
| 134 | *Rhamnus prinoides* L’Herit. | Rhamnaceae | Shrub | Gesho |
| 135 | *Rhus glutinosa*A. Rich. subsp. *glutinosa* | Anacardiaceae | Shrub | Qamo |
| 136 | *Rhus vulgaris*Meikle | Anacardiaceae | Tree | Ashkamo |
| 137 | *Ricinus communis* L. | Euphorbiaceae | Shrub | Chakima |
| 138 | *Rosa abyssinica* Lindley | Rosaceae | Shrub | Qega |
| 139 | *Rubus steudneri*Schwienf. | Rosaceae | Liana | Enjori |
| 140 | *Rumex nepalensis* Spreng. | Polygonaceae | Herb | Wisha milas |
| 141 | *Rumex nervosus* Vahl | Polygonaceae | Shrub | Ambacho |
| 142 | *Rytigynia neglecta* (Hiern) Robyns | Rubiaceae | Shrub | Dingayseber |
| 143 | *Salix subserrata*Willd. | Salicaceae | Tree | Kiya |
| 144 | *Satureja abyssinica*(Benth.) Briq.  subsp.*abyssinica* | Lamiaceae | Herb | Tosegn |
| 145 | *Satureja paradoxa* (Vatke) Engl. ex Seybold | Lamiaceae | Herb | Zikakibe like |
| 146 | *Scabiosa columbaria* L. | Dipsacaceae | Herb | - |
| 147 | *Scadoxus multiflorus*(Martyn) Raf. | Amaryllidaceae | Herb | - |
| 148 | *Schefflera abyssinica* (Hochst. ex A. Rich.) Harms | Araliaceae | Tree | Getem |
| 149 | *Scolopia theifolia* Gilg. | Flacourtiaceae | Tree | Erkudie |
| 150 | *Selaginella goudotiana* (Spring) Bizzarri | Selaginellaceae | Herb | fern |
| 151 | *Senecio* sp. | Asteraceae | Herb | - |
| 152 | *Senna singueana* (Del.) Lock | Fabaceae | Tree | Gofa |
| 153 | *Snowdenia polystachya* (Fresen.) Pilg. | Poaceae | Herb | Yekok sar |
| 154 | *Solanecio gigas* (Vatke) C. Jeffrey | Asteraceae | Shrub | Boz |
| 155 | *Solanum giganteum* Jacq | Solanaceae | Shrub | Tikuir Enbuay |
| 156 | *Solanum incanum*L. | Solanaceae | Shrub | Zerche Enbuy |
| 157 | *Sparmannia ricinocarpa* (Eckl. & Zeyh.) O. Ktze. | Tiliaceae | Shrub | Nechachiye |
| 158 | *Sporobolus africanus* (Poir.) Robyns & Tournay | Poaceae | Herb | Murig |
| 159 | *Stephania abyssinica* (Dillon & A. Rich.) Walp. | Menispermaceae | Herb | Ayt hareg |
| 160 | *Swertia abyssinica* Hochst. | Gentianaceae | Herb | Abish like |
| 161 | *Syzygium guineense*(Willd.) DC. subsp. *guineense* | Myrtaceae | Tree | Dokima |
| 162 | *Tapinanthus globiferus*(A. Rich.) Tieghem | Loranthaceae | Liana | Teketila |
| 163 | *Teclea nobilis*Del. | Rutaceae | Tree | Yenebir Tifir |
| 164 | *Tephrosla obbladensis* Chiov. | Fabaceae | Shrub | - |
| 165 | *Thalictrum rhynchocarpum* Dill. & A. Rich. | Ranunculaceae | Herb | Sire Bizu |
| 166 | *Torilis arevensis*(Hudson) Link | Apiaceae | Herb | like Dinbilal |
| 167 | *Trifolium*  sp. | Fabaceae | Herb | Maget/oxalis |
| 168 | *Trifolium decorum* Chiov. | Fabaceae | Herb | Maget |
| 169 | *Urera hypselodendron* (A. Rich) Wedd. | Urticaceae | Liana | Lankuso |
| 170 | *Urtica simensis* L. | Urticaceae | Herb | Kusha |
| 171 | *Verbascum sinaiticum* Benth. in DC. | Scrophulariaceae | Herb | Yeahya Joro |
| 172 | *Vernonia amygdalina* Del. | Asteraceae | Shrub | Girawa |
| 173 | *Vernonia leopoldi*(Sch. Bip. ex Walp.) Vatke | Asteraceae | Shrub | - |
| 174 | *Vernonia myriantha* Hook.f. | Asteraceae | Shrub | Dengorita |
| 175 | *Ximenia americana* L. | Olacaceae | Shrub | Enkoy |
| 176 | *Zehneria scabra* (Linn.f.) Sond. | Cucurbitaceae | Liana | Nech harg |
