# Supplementary material for: Relationships between topographic factors, soil and plant communities in a dry Afromontane forest patches of Northwestern Ethiopia
Source: PLoS One. 2021 Mar 12;16(3):e0247966. doi: 10.1371/journal.pone.0247966 (PMC7954303; doi:10.1371/journal.pone.0247966)
Supplement: S2 Appendix — (DOCX) [file pone.0247966.s002.docx]

**S2 Appendix.** Descriptive statistical analysis of the topography and soil data

| Environmental variable | Minimum | Maximum | Mean | Standard error |
| --- | --- | --- | --- | --- |
| Slope | 5.0 | 85.0 | 50.56 | 1.93 |
| Altitude | 1881 | 2947 | 2674.70 | 23.83 |
| Moisture content | 2.96 | 33.58 | 12.15 | 0.50 |
| Bulk density | 0.40 | 1.17 | 0.71 | 0.02 |
| Clay | 4 | 20 | 10.43 | 0.33 |
| PH | 5.93 | 7.01 | 6.45 | 0.03 |
| EC | 0.02 | 0.70 | 0.09 | 0.01 |
| OM | 0.5 | 17.930 | 8.49 | 0.36 |
| CEC | 38.2 | 89.40 | 64.19 | 1.30 |
| N | 0.03 | 0.546 | 0.37 | 0.011 |
| P | 2 | 54 | 15.76 | 1.426 |
