# Supplementary material for: Relationships between topographic factors, soil and plant communities in a dry Afromontane forest patches of Northwestern Ethiopia
Source: PLoS One. 2021 Mar 12;16(3):e0247966. doi: 10.1371/journal.pone.0247966 (PMC7954303; doi:10.1371/journal.pone.0247966)
Supplement: Attachment — Submitted filename: Answer to reviewer comments.doc [file pone.0247966.s003.doc]

# Manuscript number: PONE-D-20-31398

**Title: Relationships between topographic factors, soil and plant communities in a Dry Afromontane forest patches of Northwestern Ethiopia
 Journal: Plos One**

**Article type:** Research Article

We would like to thank the academic editor and reviewers for their efforts in reviewing the manuscript and providing valuable suggestions for improving the article. All comments and suggestions were addressed. The changes are marked in yellow color as shown in the revised manuscript. We detailed response for each comment is provided below.

Academic editor Comments:

**Comment 1:** Please ensure that your manuscript meets PLOS ONE's style requirements, including those for file naming.

**Author’s Response:** we have checked the whole PLOS ONE's style requirements, including those for file naming.

**Comment 2:** PLOS specifies that experiments, statistics, and other analyses are performed to a high technical standard; sample sizes are large enough to produce robust results; and methods are described in sufficient detail to allow another researcher to reproduce the experiment ([http://journals.plos.org/plosone/s/criteria-for-publication#loc-3](http://journals.plos.org/plosone/s/criteria-for-publication#loc-3)). As such, we ask you to amend your Methods section to provide detail on the instrumentation and methods of analysis used. Mnaufacturer information should be provided for instruments and chemicals used in the study.

**Author’s Response:** We have described the detail on the instrumentation and methods of analysis used in the revised manuscript.

**Comment 3:** In your Methods section, please provide additional information regarding the permits you obtained to collect samples for the present study. Please ensure you have included the full name of the authority that approved the field site access and, if no permits were required, a brief statement explaining why.

**Author’s Response:** We have clarified how the authority that approved the field site access in the revised manuscript. Since without permission from the conserned authority it is not possible collect both vegetion and environmental data in the study area. Please chek the revised manuscript.

**Comment 4:** Thank you for stating the following in the Acknowledgments Section of your manuscript: "The authors express their deepest thanks to Addis Ababa University for financial support. Agriculture and Rural Development Office of the Dega Damot District as well as to the respective chairpersons is also highly acknowledged."We note that you have provided funding information that is not currently declared in your Funding Statement. However, funding information should not appear in the Acknowledgments section or other areas of your manuscript. We will only publish funding information present in the Funding Statement section of the online submission form. Please remove any funding-related text from the manuscript and let us know how you would like to update your Funding Statement. Currently, your Funding Statement reads as follows: "No source of fund" Please include your amended statements within your cover letter; we will change the online submission form on your behalf.

**Author’s Response:** Again, we appreciate the reviewer’s observation. Funding related information was removed in the Acknowledgments section of the manuscript.

**Comment 5:** PLOS requires an ORCID iD for the corresponding author in Editorial Manager on papers submitted after December 6th, 2016. Please ensure that you have an ORCID iD and that it is validated in Editorial Manager. To do this, go to ‘Update my Information’ (in the upper left-hand corner of the main menu), and click on the Fetch/Validate link next to the ORCID field. This will take you to the ORCID site and allow you to create a new iD or authenticate a pre-existing iD in Editorial Manager. Please see the following video for instructions on linking an ORCID iD to your Editorial Manager account: <https://www.youtube.com/watch?v=_xcclfuvtxQ>

**Author’s Response:** Thank you very much for your excellent observation and as corresponding author I have ORCID iD based on the following video for instructions on linking an ORCID iD to my Editorial Manager account.

**Comment 6:** Please amend your list of authors on the manuscript to ensure that each author is linked to an affiliation. Authors’ affiliations should reflect the institution where the work was done (if authors moved subsequently, you can also list the new affiliation stating “current affiliation:….” as necessary).

**Author’s Response:** Again we have cheked Authors’ affiliations. As suggested by the academic editor , we have deleted Biniyam tesfaw the second previous working institution of the manuscript hereunder.

**Binyam Tesfaw^3^**

^3^School of Earth Science, Addis Ababa University, P.O. Box: 1176, Addis Ababa, Ethiopia.

E-mail: binyam.tesfaw@aau.edu.et

Deleted^: 3^Philipps-Universität Marburg, Department of Geography, Deutschhausstraße 12,35032	Marburg, Germany. E-mail: [binyam.hailu@geo.uni-marburg.de](mailto:binyam.hailu@geo.uni-marburg.de)

**Comment 7:** Please amend either the abstract on the online submission form (via Edit Submission) or the abstract in the manuscript so that they are identical.

**Author’s Response:** We have cheked either the abstract on the online submission form the abstract in the manuscript so that they are identical.

**Comment 8:** We note that Figures 1 and 2 in your submission contain map images which may be copyrighted. All PLOS content is published under the Creative Commons Attribution License (CC BY 4.0), which means that the manuscript, images, and Supporting Information files will be freely available online, and any third party is permitted to access, download, copy, distribute, and use these materials in any way, even commercially, with proper attribution. For these reasons, we cannot publish previously copyrighted maps or satellite images created using proprietary data, such as Google software (Google Maps, Street View, and Earth). For more information, see our We require you to either (1) present written permission from the copyright holder to publish these figures specifically under the CC BY 4.0 license, or (2) remove the figures from your submission:

**Author’s Response:** Our study does not need to supply a copy right notice for Figures 1 and 2. Because the location map of the study area (Fig 1) shape file data was obtained from Ethiopian Mapping Agency (<https://africaopendata.org/dataset/ethiopia-shapefiles>) is free and open to schollars. As well, the soil map of the study area (Fig 2) also the shape file data obtained from the Geographic Information System (GIS) department of the Ministry of Water, Irrigation and Electricity (MoWIE), is free and open to resercheres.

**Comment 9:** Please include your tables as part of your main manuscript and remove the individual files. Please note that supplementary tables (should remain/ be uploaded) as separate "supporting information" files.

**Author’s Response:** We have included tables as part of the main manuscript and removed the individual files.

**Comment 10:** Please include captions for your Supporting Information files at the end of your manuscript, and update any in-text citations to match accordingly. Please see our Data Availability statement you have advised "No - some restrictions will apply"

Could you please  clarify the nature of these restrictions, ie. If due to ethical or legal reasons.

**Author’s Response:** We agreed your comment and corrected data availability statement. All relevant data are within the paper and its Supporting Information files.

Reviewer #1: Comments:

**Comment 1:** It is rather interesting and refreshing to read a paper about the characterization of plant communities. This is of obvious importance in this region of Ethiopia, where plant communities have not been previously categorized. The point of this study was to document distinct plant communities in the Dega Damot District, and to relate these to various environmental factors. One of the difficulties in establishing vegetation types (communities) is natural variability. Thus, it is important to sample from a large number of habitats in order to determine how reproducible a particular community composition is, how much variability occurs within a single community type, and how much control various environmental factors exert on community structure.Based on the description of the sampling effort, it is difficult for me to determine how much sampling actually occurred within a community type.

**Author’s Response:** We have agreed your sugessions, One of the difficulties in establishing vegetation types (communities) is natural variability. The idea of the plant community was hotly debated by early plant ecologists and is still a matter of contention today. Two American ecologists, Clements and Gleason expressed the extreme viewpoints. Clements (1916, 1928) saw plant communities as clearly recognizable and definable entities, which repeated themselves with great regularity over a given region of the Earth’s surface. Gleason (1917, 1926, and 1939) saw all plant species distributed as a continuum. He argued that plants respond to variations in environmental factors, and those factors vary continuously in both space and time. As a result, the combination of plant species found at any given point on the earth the surface is unique. For instance, in this study all the measured environmental variables are not correlated to in establishing vegetation types (communities) and it can be other environmental variables may influence the community formation.

**Comment 2**: The authors refer to “forest patches”, but just what these are is not clear. “Sampling sites were arranged along transects from the forest patches”. There were 6 transects in both in Shangi Derke and Bunsie patches. There were 4 transects in the Aradie patch. And there were 3 transects in both Masikana and Samana patches. Were these patches already known to contain distinct plant communities so the level of replication within a community type could be controlled? If not, how did the authors control the level of replication within a communitytype?

**Author’s Response:** Due to habitat loss and fragmentation previous contiguous forest is reduced smaller patches of forest. Thus, we used 5 smaller patches of forests in the study area for classification of vegetion (Plant community types). We pooled the vegetion data obtained from all the 5 forest patches for determination of vegetion types except one forest patch ( Samana) the rest did not show distinct plant communities. The plant community can be defined as the collection of plant species growing together in a particular location that shows a definite association or affinity with each other.

**Comment 3:** By my reading, it appears that 86 plots were sampled in each of the 5 patches, for a total of 430 plots. Is that true? The authors wrote “The first transect and plot were selected purposely which are free from any anthropogenic disturbances.” How was that determined? Does that mean that some plots were disturbed? If so, wouldn’t disturbance influence the structure of the plant community, and wouldn’t this bias our understanding of these communities?

**Author’s Response:** We have rewritten the paragraph to improve its clarity. A total of 86 sampling plots of each measuring 900m^2^ (30 m × 30 m) were taken from all 5 forest patches. Of which, 29 from Shangi Derke, 32 from Bunsie, 10 from Aradie , 9 from Masikana Medihaniyalem and and 6 from Samana forest patches were sampled. The first transect and plot were selected purposely at one side of the forest by avoiding the forest edge.

**Comment 4**: There were apparently 5 smaller plots per larger plot for herbaceous plant sampling for a total of 2,150 small plots. Is that true? NO Also, please specify when the smaller plots were sampled. Is it possible for sampling time to affect the herbaceous species that were observable? Please specify. Because the spatial and temporal aspects of the vegetation and soil sampling schemes were not clear to me, perhaps a figure illustrating them would be helpful.

**Author’s Response:** When a study mainly focuses on only woody species composition and diversity, research has mainly ignored studies on diversity and not included herbaceous species and rare species which are important to the understanding of a comprehensive list of plant species diversity in the study area. Therefore we have included the herbaceous species. Five smaller subplots of 2 *2 m^2^, four at the corner, and one at the center of the main plot were established for herbaceous plant species collection. If you think that the estimation of herbaceous species from the field plots is difficult and time-consuming, you are correct. That is why vegetation ecologists have developed different methods. In our study the overall cover of herbaceous species estimated by the average of individual cover values of species taken from the subplots (4 m^2).^ (The herbaceous data were taken from five replicates (from corneres and one from the center) and only one sample was taken for analysis (total 86) not cover estimation was done for ,150 small plots.

**Comment 5**. I am not sure how the cluster analysis was performed. The authors wrote that clustering was performed based on attributes and floristic similarities. What is meant by “attributes”? Does that include physical traits of the habitat? Please be more specific about the exact variables that were used in the cluster analysis.

**Author’s Response**: Classification by means of cluster analysis is the most common multivariate technique used in ecological works. In this study, a hierarchical (agglomerative) cluster analysis was performed using the free statistical software R. The classification was done by using the cover abundance value estimate of each species included in the analysis. Cluster analysis used to classify a set of observations (plots) in to groups based on their floristic similarities. Therefore for this study the data matrix contained 86 quadrats/plots and 164 plant species were grouped using hierarchical cluster analysis, ie.determine plots that can be classified into the same groups based on the species abundance data

**Comment 6:** Table 7 indicates that several of these variables are significant, but the authors should be cautious as none of them account for much variability (sums of squares are all low). This should be indicated in the text.

**Author’s Response:** Thank you for this excellent observation. We described several of these variables are significant but none of them account for much variability except elevation. For instace, A high sumof squeres would indicate a lot of variability in the data, while a low sum of squeres ( most of the measurement close to mean) would indicate a low amount of variability. We have indicated in the revised manuscript.

Reviewer #2:

**Comment:** This work by Birhanu et al. describes the results of comprehensive vegetation surveys across 86 sites in Northwestern Ethiopia. They evaluated how climatic and topoedaphic variation determine patterns of plant community composition and species abundance across the region. They highlighted clusters of community types based on hierarchical analysis and a series of standard community composition metrics. These species-rich sites were compared to other high diversity forest areas in the region, as well as to forests in Cameroon. The methods used in this study were appropriate and rigorously applied, and the conclusions they drew in their analysis are supported by their results. This well-written work appropriately characterizes the roles of soils, climate and topography in driving community composition across the region, and I recommend it be accepted for publication.

**Author’s Response:** Thank you very much for your positive feedback. We found your comments extremely helpful.
